# Efficacy and safety of combined Chinese and Western medicine therapy for hypertensive intracerebral hemorrhage: A systematic review and meta-analysis of randomized controlled trials

**DOI:** 10.1097/MD.0000000000044632

**Published:** 2025-10-10

**Authors:** Lixiaotian Zong, Desheng Zhou, Yan Zeng, Zhong Li, Xuqing Zhou, Huan Dong, Chun Guo

**Affiliations:** a Experimental Laboratory Center, The First Affiliated Hospital of Hunan University of Chinese Medicine, Changsha, Hunan, China.

**Keywords:** Chinese and Western medicine, efficacy, hypertensive cerebral hemorrhage, meta-analysis, systematic review

## Abstract

**Background:**

Hypertensive intracerebral hemorrhage (HICH) is a critical neurological emergency associated with high morbidity and mortality. Conventional Western medical therapies – such as antihypertensive treatment, intracranial pressure reduction, and supportive care – provide symptomatic relief but remain limited in promoting hematoma absorption, neurological recovery, and long-term functional outcomes. Traditional Chinese medicine (TCM) has shown clinical potential in enhancing neurological repair and improving prognosis. Given their complementary strengths, combined Chinese and Western medicine may represent a more comprehensive treatment strategy. However, the overall efficacy and safety of such integrative approaches remain uncertain due to inconsistent findings and lack of high-quality evidence.

**Methods:**

A comprehensive search of CNKI, VIP, CBM, PubMed Embase, and Cochrane Library was conducted from 2010 to March 2024. Randomized controlled trials (RCTs) comparing combined therapy with Western medicine alone were included. The primary outcome was overall clinical effective rate. Secondary outcomes included National Institutes of Health Stroke Scale (NIHSS) score, Glasgow Coma Scale (GCS) score, hs-CRP level, adverse events and mortality. Data extraction, risk-of-bias assessment using the Cochrane tool and statistical analyses were independently performed by 2 reviewers. Meta-analysis was conducted with fixed- or random-effects models depending on heterogeneity, while sensitivity, subgroup, publication bias and trial sequential analyses (TSA) were also performed.

**Results:**

Eighteen RCTs involving 1644 participants were analyzed. Compared with Western medicine alone, combined therapy significantly improved clinical effective rate (OR = 4.35, 95% CI [3.08–6.13], *P* < .00001), NIHSS score (MD = −2.95, 95% CI [−2.29 to −1.95], *P* < .00001), GCS score (MD = 1.95, 95% CI [1.61–2.28], *P* < .00001), and reduced hs-CRP levels (MD = −1.31, 95% CI [−1.44 to −1.19], *P* < .00001). No significant difference was observed in adverse events (OR = 0.81, *P* = .43). Mortality was reported in only one trial and showed no difference between groups (*P* > .05). TSA supported the sufficiency and robustness of evidence for the primary outcome.

**Conclusion:**

The therapeutic effect of combined traditional Chinese and Western medicine in treating HICH is better than that of Western medicine alone.

## 1. Introduction

Intracerebral hemorrhage (ICH) is a disease in which blood enters the brain parenchyma or ventricles due to rupture of blood vessels in the brain that can cause neurological damage.^[1]^ ICH is a disease with a high mortality and morbidity rate, accounting for 10% to 20% of all strokes, and the incidence rate is as high as 30% in Asian and African countries.^[2]^ The risk of cerebral hemorrhage in patients with hypertension is 3 to 5 times that of people with normal blood pressure, and hypertensive cerebral hemorrhage is the most common cause which accounts for 60% to 80% in ICH.^[3]^ And hypertensive intracerebral hemorrhage (HICH) is one of the types of stroke with the highest mortality rate, with a 30-day mortality rate of up to 40% and a 1-year mortality rate of more than 50%.^[4]^ In addition, 60% of surviving may be left with varying degrees of neurological dysfunction, such as hemiplegia, language disorders and cognitive impairment.

The most common treatments for HICH are conservative treatment, surgical treatment and rehabilitation treatment.^[5]^ Conservative treatment is to eliminate the hematoma of ICH patients through blood pressure management, reducing intracranial pressure and hemostasis.^[6]^ However, conservative treatment usually cannot directly remove the hematoma, and the long-term existence of the hematoma may cause secondary brain injury. There are 3 main surgical treatment methods, namely craniotomy hematoma removal, minimally invasive hematoma aspiration and ventricular drainage.^[7]^ But the large trauma of craniotomy may aggravate brain injury, minimally invasive surgery cannot completely clear the hematoma, and ventricular drainage is only suitable for ventricular blood accumulation and can’t remove brain parenchymal hematoma. Rehabilitation treatment is only for patients who have received treatment and have residual functional deficits, and the rehabilitation effect is usually limited.^[8]^

In recent years, traditional Chinese medicine (TCM) has shown unique advantages in the treatment of HICH and has become a hot topic in clinical research.^[9–11]^ TCM treatment of HICH has the characteristics of multitarget comprehensive regulation. It can improve the pathological state of HICH and improve the therapeutic efficacy of drugs through multiple pathways of promoting blood circulation and removing blood stasis, clearing away heat and detoxifying.^[9]^ Studies have found that *Panax notoginseng* and *Salvia miltiorrhiza* can promote hematoma absorption, reduce secondary injury and the incidence of neurological dysfunction.^[12]^ Moreover, promoting blood circulation and removing blood stasis is an important means of TCM treatment of HICH, such as Xuefu Zhuyu Decoction^[13]^ and *Salvia miltiorrhiza* injection.^[14]^ In addition, the Chinese medicinal materials *Astragalus membranaceus*,^[15]^ *Carthamus tinctorius*^[16]^ and *Ligusticum sinense*^[17]^ can inhibit inflammation and reduce brain edema by regulating the expression levels of inflammatory factors which can improve the recovery effect of patients.

Although traditional Western medicine can stabilize the condition and reduce complications in the acute phase of HICH, it also has certain limitations and disadvantages. TCM has significant advantages in the treatment of HICH, such as promoting hematoma absorption, reducing secondary injury, and improving neurological function recovery.^[9]^ However, the current research of combined Chinese and Western medicine therapy for HICH still has the problem of unclear mechanism of action and lack of large-scale clinical trials. Therefore, our study systematically evaluated and meta-analyzed RCTs of T combined Chinese and Western medicine therapy for HICH to provide more reliable clinical evidence for TCM treatment of ICH.

## 2. Methods

### 2.1. Literature search

A comprehensive literature search was conducted in PubMed, Cochrane Library, Embase, Web of Science, China National Knowledge Infrastructure (CNKI), National biomedical literature service system (SinoMed), Chinese Science and Technology Journal Database (VIP), and Wanfang databases from January 2010 to March 2024. We also searched gray literature (such as unpublished conference abstracts, clinical trial registries and government reports) to reduce publication bias. This study used a combination of standardized subject headings and free-text terms from the databases with English and Chinese to improve search precision and ensure coverage of new studies that have not been indexed, the search keywords were “Hypertensive,” “Intracerebral hemorrhage,” “Western medicine,” “Chinese and Western medicine,” “Chinese medicine” and “Traditional Chinese medicine “.

The inclusion criteria for the papers: the article should be a randomized controlled clinical trial of TCM combined with treatment of hypertensive ICH. ICH patients should meet the criteria of the “Guidelines for the Diagnosis and Treatment of ICH in China (2019)” published in 2019,^[18]^ and the diagnosis of hypertension should meet the judgment criteria of the World Health Organization (WHO)/International Society of Hypertension (ISH) on hypertension management^[19]^; the experimental group must be treated with Chinese and Western medicine, and Chinese medicine treatment must be herbal medicine; the control group should be treated with placebo or single Western medicine treatments; the primary outcome measures were clinical efficacy and neurological deficit score; the secondary outcome measures were mortality and adverse events at the end of long-term follow-up of at least 2 weeks.

The exclusion criteria for the papers: not randomized controlled trials (RCTs); inability to obtain accurate research data for subsequent analysis; lack of a control group; systematic reviews, meta-analyses and case reports.

### 2.2. Data screening

All downloaded literature was screened by 2 researchers (LZ and DZ) in terms of titles and abstracts, and those that did not meet the inclusion criteria were excluded. It was not possible to determine whether the inclusion criteria were met in the title and abstract of the literature, and the researchers needed to read the full text to confirm whether they met the inclusion criteria. There were inconsistencies in the screening results of the 2 researchers, and a third professional researcher was required to make a judgment. The 2 researchers extracted the methodological characteristics, demographic baseline, diagnostic criteria, intervention measures and outcome measures of each literature.

### 2.3. Quality assessment

The 2 researchers evaluated the quality of the literature based on the risk of bias (ROB) assessment tool in the Cochrane Reviewers Handbook 6.1 and checked the results with each other.^[20]^ The assessment content includes 7 aspects, namely random sequence generation (selection bias), allocation concealment (selection bias), blinding of participants and personnel (performance bias), blinding of outcome assessment (detection bias), incomplete outcome data (attrition bias), selective reporting (reporting bias) and other bias (such as bias related to special study designs, baseline differences and data falsification.). The included literature was rated as “low risk,” “uncertain” or “high risk.” If there is a disagreement in the evaluation results of the 2 researchers, the third researcher will negotiate and make a decision.

### 2.4. Statistical analysis

This study was conducted using Review Manager 5.3 for meta-analysis. The analysis was conducted using trial sequential analysis (TSA) software^[21]^ developed by the Copenhagen Trial Unit (https://www.ctu.dk/tsa/). The primary outcome (clinical effective rate) was used for TSA. For dichotomous outcomes, we inputted the number of events and total sample size from each included RCT. Parameters were set as follows: two-sided α = 0.05, power (1 − β) = 0.80 and a relative risk reduction based on the pooled effect size from the conventional meta-analysis (OR = 4.35). The effect size of dichotomous data was expressed as odds ratio (OR) and its 95% confidence interval (CI), while continuous variables were calculated using weighted mean difference (MD). If there were differences in measurement units among studies, standardized mean difference (SMD) was used to adjust. All variables were reported as effect size with 95% CI. In addition, heterogeneity among studies was assessed using Chi^2^ test and *I*^2^ statistic, *P* ≥ .05 or *I*^2^ ≤ 50% indicating low heterogeneity that a fixed-effect model was used, otherwise, a random-effect model was used for analysis.

## 3. Result

### 3.1. Literature screening

**Figure 1.**
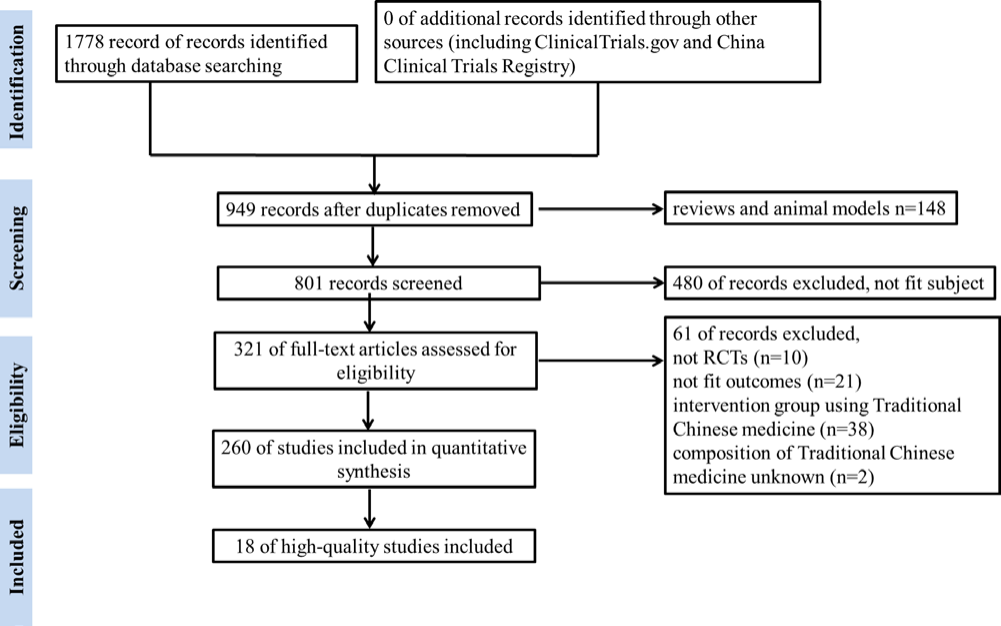
Flow chart of literature screening.

This study retrieved a total of 1778 articles from various databases. After deleting 829 duplicate articles, 949 articles were available for subsequent screening (Fig. 1). There were 148 reviews, systematic reviews and animal experiments, 480 articles with inconsistent research content, 61 trials with inconsistent intervention and control methods, and 260 studies included in quantitative synthesis after screening. Finally, 18 articles that met the inclusion criteria of this study were included in this meta-analysis after abstract and full text screening.

### 3.2. Study characteristics

All 18 clinical studies^[22–39]^ were RCTs, with a total of 1644 cases included, including 825 cases in the experimental group and 819 cases in the control group, with an average total sample size of 92 cases (Table 1). The control group received conventional treatment of Western medicine, while the experimental group received a combination of Chinese and Western medicine. The efficacy indicators included effective rate, volume of cerebral hemorrhage, stroke neurological deficit score formulated by the 4th National Cerebrovascular Disease Academic Conference, National Institutes of Health Stroke Scale (NIHSS) score, Glasgow Coma Scale (GCS) score, high-sensitivity C-reactive protein (hs-CRP) and adverse events.

**Table 1:** Basic information of the literature on the treatment of hypertensive cerebral hemorrhage with integrated traditional Chinese and Western medicine included in the study.

Study	Sample size T/C	Intervention measure		Treatment/week	Therapeutic index
		T	C		
Zeng et al, 2012	40/40	CI + Qinggan Decoction	MT	1	35
Chen, 2017	36/36	CI + Blood-activating and stasis-removing herbs	MT	1	147
Fu and Li, 2011	31/30	CI + Heat-clearing, cooling, blood-stopping, bowel-clearing and heat-clearing medicine	MT	2	1
Ju et al, 2018	46/46	CI + Buyang Huanwu Decoction	MT	2	1456
Li et al, 2011	88/85	CI + Xingshen Kaiqiao Liquid	MT	4	27
Li et al, 2015	45/45	CI + Chinese medicine Huayu Ditan Decoction	MT	2	12,467
Liu et al, 2011	26/25	CI + Di Decoction in acute phase, Tianma Gou Teng Yin in stable phase, Buyang Huanwu Decoction in recovery phase	MT	4	12
Liu, 2014	30/30	CI + Fu-clearing and heat-clearing medicine	MT	2	12
Ma and Yan, 2011	29/29	CI + Tongfu Huoyu Decoction	MT	3	1
Ruan, 2018	44/44	CI + Huoxue Ditan Decoction in acute phase, Buyang Huanwu Decoction in stable phase	MT	4	1
Shen and Luo, 2015	33/32	CI + Xuefu Zhuyu Decoction	MT	4	12
Yang, 2019	45/45	CI + Buyang Huanwu Decoction	MT	2	1245
Yin and Wei, 2014	41/41	CI + Buyang Huanwu Decoction	MT	3	1
Zhou et al, 2019	50/50	CI + Acute phase blood circulation and blood stasis medicine, stable phase blood-activating and stasis-removing herbs	MT	4	17
Zhou et al, 2016	40/40	CI + Buyang Huanwu Decoction	MT	2	14,567
Jiang and Wang, 2021	41/41	CI + Blood-activating and stasis-removing herbs	MT	2	34
Ye, 2023	40/40	CI + Blood-activating and stasis-removing herbs	MT	2	1346
Li et al, 2016	120/120	CI + Blood-activating and stasis-removing herbs	MT	12	147

T is the observation group; C is the control group; CI is the control measure; MT is the conventional treatment of Western medicine (regulating intracranial pressure and blood pressure, nourishing cranial nerves, preventing and controlling cerebral edema, correcting electrolyte and acid-base disorders, treating underlying diseases and complications of various systems, nutritional support). The efficacy indicators included effective rate, volume of cerebral hemorrhage, Stroke Neurological Deficit Score formulated by the 4th National Cerebrovascular Disease Academic Conference, National Institutes of Health Stroke Scale (NIHSS) score, Glasgow Coma Scale (GCS) score, high-sensitivity C-reactive protein (hs-CRP), adverse events.

### 3.3. Assessment of ROB

**Figure 2.**
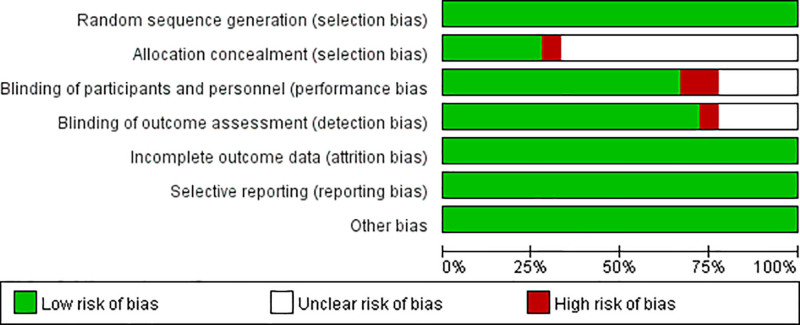
Risk of bias graph for included RCTs. RCTs = randomized controlled trials.

This study used the Cochrane bias risk tool to assess the ROB in 18 studies from 7 aspects (Fig. 2). All 18 studies generated random sequences through random number tables, and all patients had complete clinical data. The results of these trials did not show selective bias, and no other bias risks were found. Therefore, these studies were judged to be low risk in 4 aspects. Inter-rater reliability for the ROB assessment was calculated using Cohen kappa coefficient, yielding a value of 0.82, which indicates good agreement between the 2 reviewers. Based on GRADE assessment, the quality of evidence for the primary outcome (clinical effective rate) was rated as high, supported by a large pooled sample size, consistent results across studies (*I*^2^ = 0%), and overall low ROB (Table S1, Supplemental Digital Content, https://links.lww.com/MD/Q202). The evidence quality for secondary outcomes such as NIHSS and GCS scores was moderate, as the studies showed clinically meaningful improvements but presented some heterogeneity and imprecision. The certainty of evidence regarding adverse events and mortality was rated as low, due to limited number of events and small number of reporting studies.

**Figure 3.**
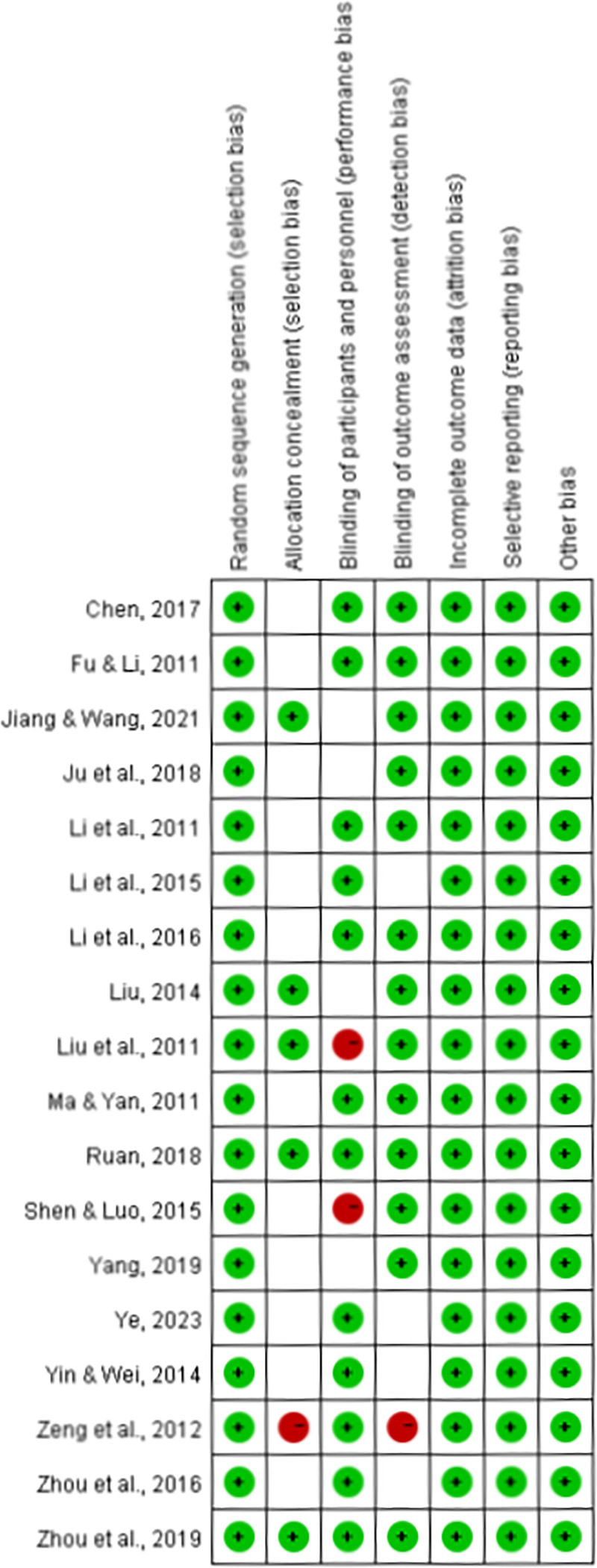
Risk of bias summary for included RCTs. RCTs = randomized controlled trials.

In allocation concealment, allocation concealment was considered low risk in 5 studies because they used opaque envelopes to randomly allocate patients (Fig. 3). The remaining trials did not mention allocation concealment. 1 trial was assessed as high risk because they used a random number table to allocate patients. In blinding of participants and personnel, 12 trials clearly stated how participants and personnel were blinded which was considered low risk. However, 4 trials were considered unclear risk because the trials described the personnel who carried out the blinding of the trial and there was insufficient evidence to confirm that the participants were also blinded. 2 trials were assessed as high risk because they did not provide enough information on whether personnel or participants were blinded to the treatment measures. In blinding of outcome assessment, 13 trials were considered low risk because they mentioned blinding of outcomes. 1 trial was judged as high risk because they did not state whether the outcomes were blinded.

## 4. Efficacy outcomes

### 4.1. Effective rate

**Figure 4.**
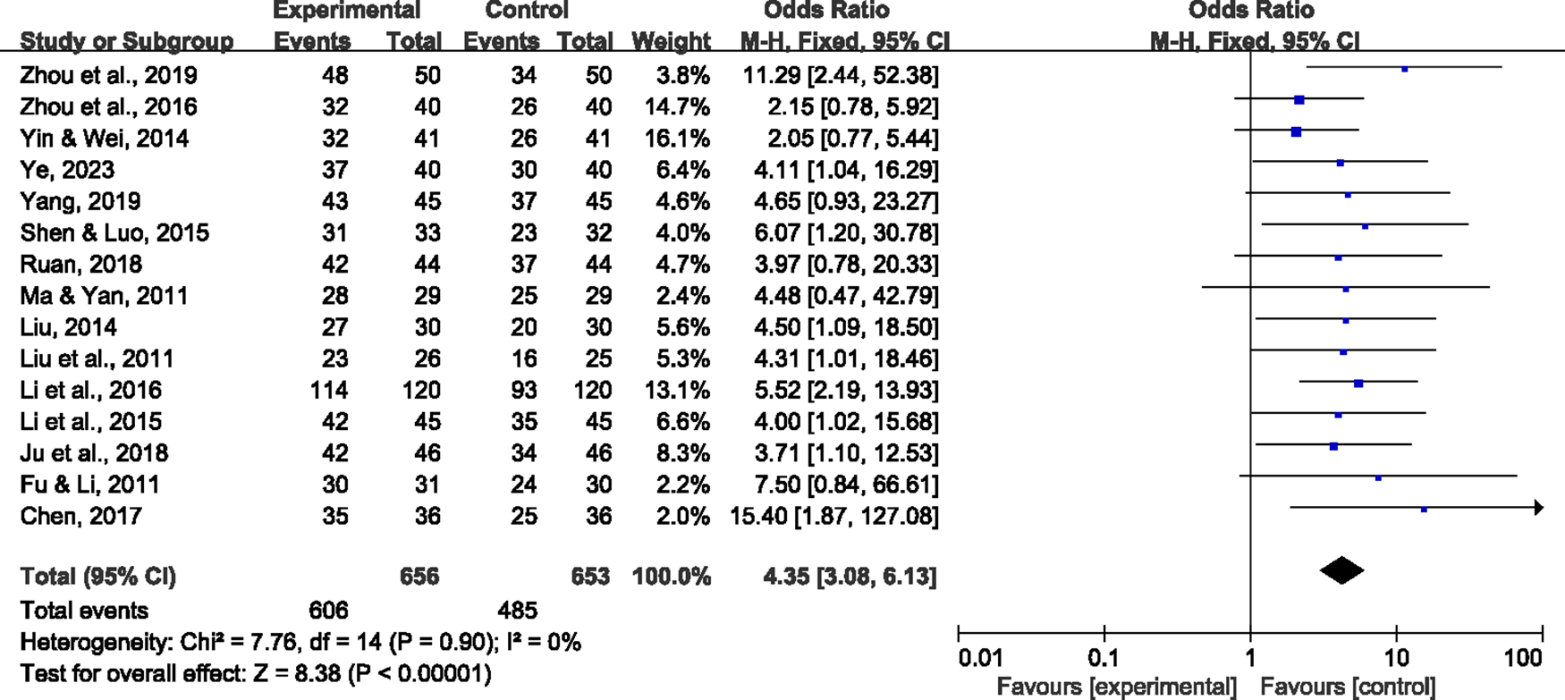
The forest plot demonstrated the clinical efficacy of combined Chinese and Western medicine therapy for HICH. HICH = hypertensive intracerebral hemorrhage.

15 studies used the effective rate of TCM combined treatment of HICH as the main clinical treatment indicator (Fig. 4). The combined effective rate of each trial study was *P* = .90, *I*^2^=0%, indicating that there was no significant heterogeneity between the studies, so the fixed-effect model was used for data pooling analysis. The results showed that the effective rate of the TCM combined treatment group was significantly higher than that of the control group (OR = 4.35, 95% CI = [3.08–6.13]), with a combined, and the difference was statistically significant (*P* < .00001). This shows that TCM combined treatment has significant advantages in improving the clinical efficacy of HICH patients, and helps to improve the patient’s neurological function recovery and overall prognosis.

### 4.2. NIHSS score

**Figure 5.**
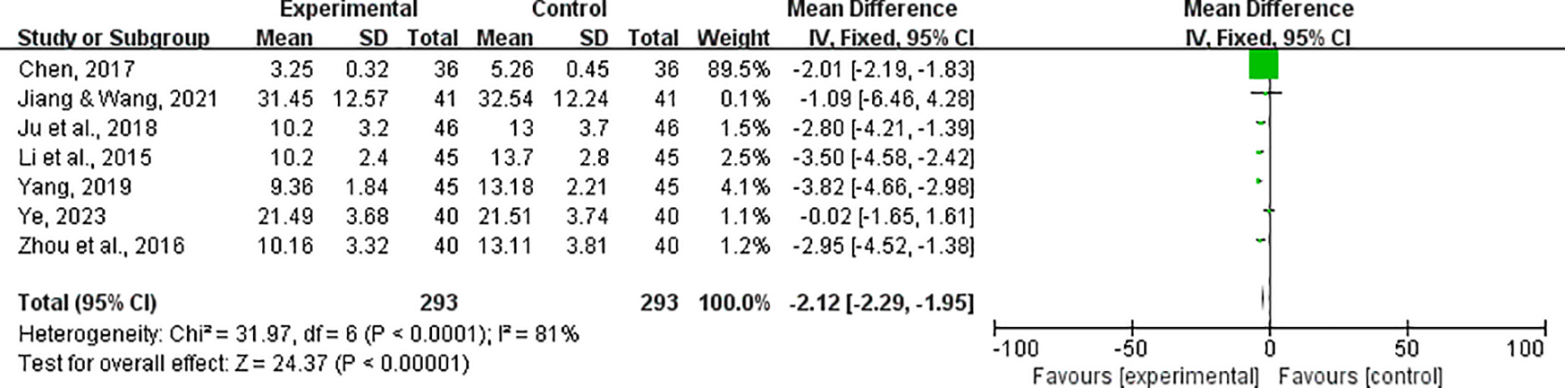
The forest plot demonstrated improvement of NIHSS by combined Chinese and Western medicine therapy for HICH. HICH = hypertensive intracerebral hemorrhage.

Seven RCT trials analyzed the NIHSS score of HICH patients treated with integrated Chinese and Western medicine as a secondary treatment indicator (Fig. 5). The study found that there was heterogeneity in the consistency of the trial results (*P *< .01, *I*^2^ > 50%), and a random-effects model was used to evaluate the improvement of neurological deficits. The synthesis of these trial results showed that compared with the control group, the NIHSS score of HICH patients receiving integrated Chinese and Western medicine treatment was significantly improved (MD = −2.95, 95% CI [−2.29 to −1.95], *P *< .00001).

### 4.3. GCS score

**Figure 6.**
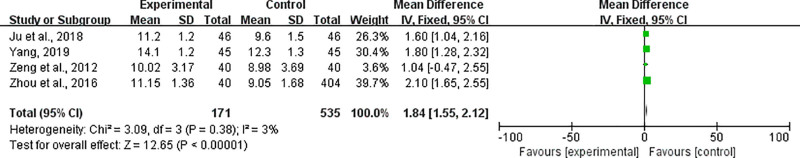
Forest plot of GCS scores in literature related to the treatment of hypertensive cerebral hemorrhage with combined Chinese and Western medicine therapy. GCS = Glasgow coma scale.

There are 4 studies analyzing the GCS scores of HICH patients treated with TCM, with a total of 342 patients (Fig. 6). The combined statistical analysis of the results of each study found that there was no significant heterogeneity between the studies (*P* = .55, *I*^2^ = 0%), so the fixed-effect model was used for analysis. The results showed that the GCS score of the combined traditional Chinese and Western medicine treatment group was significantly higher than that of the Western medicine treatment group after treatment (MD = 1.95, 95% CI [1.61–2.28], *P* < .00001), which also shows that the consciousness of the patients in the experimental group is significantly better than that in the control group.

### 4.4. hs-CRP

**Figure 7.**
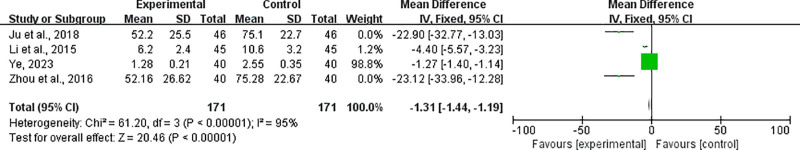
Forest plot of hs-CRP in literature related to the treatment of hypertensive cerebral hemorrhage with combined Chinese and Western medicine therapy. hs-CRP = high-sensitivity C-reactive protein.

Four of the included studies reported hs-CRP as a secondary treatment indicator, and a total of 342 patients were included (Fig. 7). The study found that the heterogeneity of hs-CRP in the control and experimental groups of each study result was large (*P < *.00001, *I*^2^ = 95%). Further analysis using the random-effects model found that the hs-CRP of HICH patients treated with combined Chinese and Western medicine was significantly lower than that of the Western medicine treatment group (MD = −1.31, 95%Cl = [−1.44, −1.19], *P < *.00001).

### 4.5. Adverse events

**Figure 8.**
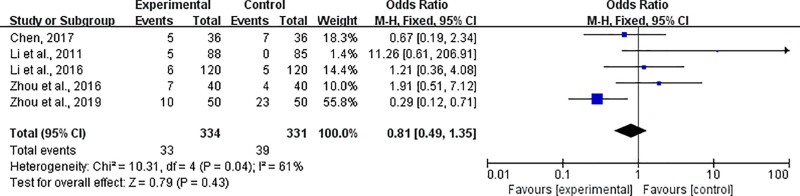
Forest plot of adverse events related to the treatment of HICH with combined Chinese and Western medicine therapy. HICH = hypertensive intracerebral hemorrhage.

Among the included studies, 5 studies included adverse reactions in their outcome indicators, including fever, dizziness, nausea, irritability, upper abdominal discomfort, insomnia, diarrhea, coma, itching on the body surface and renal function impairment (Fig. 8). In one of the studies, no adverse events occurred in the control group, but 5 adverse events occurred in the experimental group. There was a certain degree of heterogeneity among the adverse reaction rate results (*P* = .041, *I*^2^ = 61%). Eliminating individual studies one by one had no significant effect on the heterogeneity of the overall outcome. The random-effects model was used (OR = 0.81, 95% C1 = [0.49, 1.35] indicating that the adverse reaction rate in the experimental group was significantly different from that in the control group (*P* = .43).

### 4.6. Mortality rate

Among these 18 studies, only the study by Li et al (2016) reported mortality. In this study, there were 2 deaths in both the control group and the experimental group (*P* > .05). This further demonstrated the safety of combined TCM in the treatment of HICH patients.

### 4.7. Trial sequential analysis

**Figure 9.**
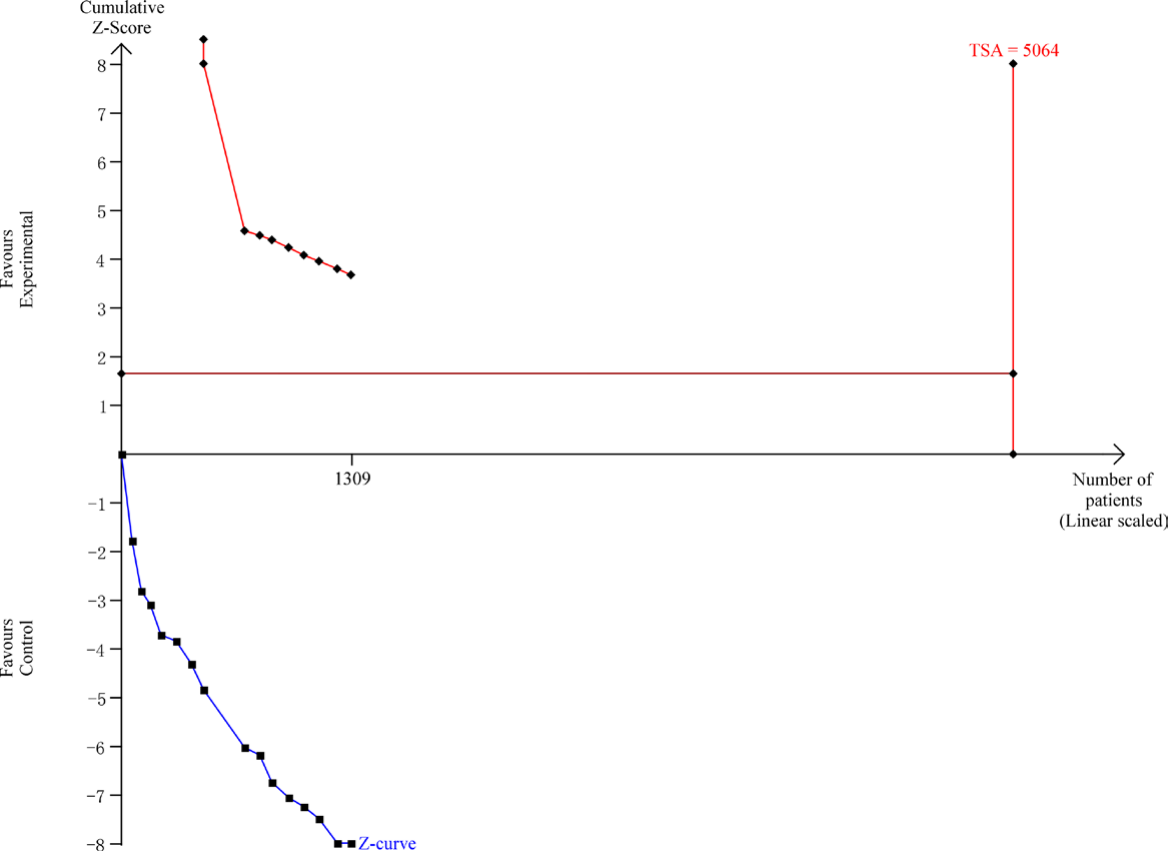
TSA of clinical effective rate with combined Chinese and Western medicine therapy for HICH. TSA = trial sequential analysis, HICH = hypertensive intracerebral hemorrhage.

To evaluate the robustness of the meta-analysis findings and control for potential random errors, a TSA was conducted using data from 15 RCTs reporting the clinical effective rate. The TSA demonstrated that the cumulative number of participants (n = 1309) accounted for only 25.9% of the required information size (RIS = 5064) (Fig. 9), indicating a substantial shortfall in sample size to confirm the intervention effect with sufficient statistical power. The cumulative *Z*-curve did not cross the conventional boundary for statistical significance nor the TSA monitoring boundary for benefit. This suggests that although the pooled OR from the conventional meta-analysis favored the experimental group (OR = 4.35, 95% CI = 3.08–6.13, *P* < .00001), the current evidence may still be prone to type I error due to insufficient data. Furthermore, the trajectory of the *Z*-curve showed an initial trend toward benefit but gradually plateaued, indicating a potential overestimation of the intervention effect in early trials. The required information size was not reached, and the sequential boundaries were not crossed which can imply that the evidence remains inconclusive under TSA.

The TSA suggests that while conventional meta-analysis results show statistically significant benefit of combined Chinese and Western medicine over Western medicine alone, these findings are not yet sufficiently supported by cumulative evidence. Additional large-scale, high-quality RCTs are required to confirm the observed treatment effect and ensure the reliability of the conclusions.

### 4.8. Exploration of heterogeneity and publication bias

For all outcomes with substantial heterogeneity (*I*^2^ > 50%), such as NIHSS score (*I*^2^ > 50%), hs-CRP (*I*^2^ = 95%), and adverse events (*I*^2^ = 61%), subgroup and sensitivity analyses were conducted to explore potential sources of heterogeneity. Subgroup analyses were based on factors including treatment duration, sample size, and types of Chinese herbal medicines used. These analyses suggested that differences in treatment duration and herbal formulations might contribute to observed heterogeneity. Sensitivity analyses were performed by sequentially excluding each study to evaluate the robustness of the pooled estimates. Results remained consistent without significant changes in effect size or heterogeneity, supporting the stability of the findings.

Publication bias was assessed by visual inspection of funnel plots and Egger test for the primary outcome (clinical effective rate) (Fig. S1, Supplemental Digital Content, https://links.lww.com/MD/Q203). The funnel plots were largely symmetrical, and Egger test did not detect significant publication bias (*P* > .05), indicating low likelihood of bias from selective publication.

## 5. Discussion

HICH is one of the most common types of cerebral hemorrhage and one of the serious complications of hypertension.^[1]^ The main clinical features of HICH are acute headache, dizziness, limb paralysis, pupil abnormalities and even loss of consciousness.^[40]^ Moreover, studies have found that 30% of patients with hypertensive cerebral hemorrhage suffer from physical disability after treatment, which seriously affects the quality of life of patients.^[41]^ The treatment of hypertension in Western medicine is to control intracranial pressure by hemostasis and hypotension.^[42]^ TCM uses promoting blood circulation to remove blood stasis, and nourishing blood as treatment methods. TCM pays more attention to the overall conditioning of HICH patients, rehabilitation during the recovery period and prognosis management. Due to the different advantages of traditional Chinese and Western medicine in treating HICH, more and more clinical studies are combining traditional Chinese and Western medicine to treat HICH.^[9,43,44]^

The findings of this study are consistent with those of Xu et al (2017),^[42]^ an earlier systematic review and meta-analysis, both demonstrating that integrated Chinese and Western medicine can improve clinical outcomes in hypertensive intracerebral hemorrhage (HICH). However, Xu et al included RCTs published between 2000 and 2016, with a total of 10 studies, whereas the present study analyzed 18 RCTs published between 2010 and 2024, expanding both the sample size and the temporal coverage. Moreover, while Xu et al evaluated treatment efficacy based on clinical response rate, neurological deficit improvement, reduction in hematoma volume, and adverse events, our study employed NIHSS and GCS scores for a more comprehensive assessment of neurological recovery, and additionally analyzed levels of hs-CRP to explore the effects of the combined treatment on HICH.

The results of this study show that Chinese medicine combined with Western medicine internal medicine treatment can significantly improve the clinical efficacy of HICH, promote neurological function recovery and clear consciousness, and there is no significant difference in the occurrence of adverse events between the 2 groups, which is more effective than simple Western medicine basic treatment. Except for the studies of Zhou et al (2016)^[36]^ and Yin and Wei (2014)^[34]^ which showed that the efficacy of combined traditional Chinese and Western medicine in the treatment of hypertensive cerebral hemorrhage (HICH) was <90%, all other studies had treatment efficiencies higher than 90%. In Zhou et al’s study, the treatment efficiency of the control group (Western medicine) was 90.00%, slightly higher than that of the combined treatment group (87.50%). In Yin et al’s study, the control group showed a treatment efficiency of 63.41%, compared to 78.64% in the combined group. Fu et al (2011)^[24]^ showed the best treatment efficiency for the combined use of Chinese and Western medicine in the treatment of HICH with an effective rate of 96.77%, compared to 86.70% in the control group. This result indicates that the combination of Chinese and Western medicine can improve the treatment efficiency of patients with HICH treated with Western medicine.

The most commonly used Chinese medicine auxiliary drugs for HICH patients are blood-activating and stasis-removing herbs.^[45]^ Cerebral arteriosclerosis and hypertension are the main causes of cerebral hemorrhage. The advantages of drug therapy for promoting blood-activating and stasis-removing herbs can’t aggravate cerebral arteriosclerosis and increase blood pressure.^[46]^ Studies have found that blood-activating and stasis-removing herbs participate in the regulation of systemic blood circulation to maintain normal circulatory function.^[9]^ Blood-activating and stasis-removing herbs can also improve capillary permeability to enhance phagocyte function, accelerate fibrinolysis and promote the establishment of cerebral vascular collateral circulation, which can promote the absorption of cerebral hematoma and the elimination of cerebral edema and improve the ischemia of brain cells around cerebral hematoma to achieve brain cell protection.^[47]^

NIHSS score and GCS score are both commonly used clinical neurological assessment tools to evaluate the severity of neurological impairment, especially in patients with HICH and stroke.^[48]^ Single Western medicine treatment methods often have certain limitations in the treatment of HICH. The various active ingredients in TCM have anti-inflammatory, antioxidant and microcirculation-improving effects, which can promote the repair of brain tissue and the recovery of neurological function.^[49]^ Studies have found that some TCMs with blood circulation and stasis-removing effects can reduce blood viscosity, improve brain blood circulation, reduce hematoma compression and brain edema after cerebral hemorrhage, such as *Panax notoginseng* and *Salvia miltiorrhiza*.^[14]^ Among the 18 studies in this study, 10 studies selected TCMs with blood circulation and stasis-removing effects as auxiliary drugs, and these studies found that the combination of Chinese and Western medicines can significantly improve the treatment effect of HICH patients.

The 18 Chinese medicines for the treatment of HICH with combined Chinese and Western medicine in this study can be divided into 5 categories, namely tonifying and blood-activating, blood-activating and stasis-resolving, heat-clearing and purgative, spirit-refreshing and orifice-opening, and compound drug. Among the 18 studies in this study, 10 studies used blood-activating Chinese medicine as auxiliary drugs. Moreover, the therapeutic effect of blood-activating Chinese medicine as auxiliary drugs for HICH treated with Western medicine in this study was better than that of other drugs. Therefore, in the future development of auxiliary drugs for Western medicine to treat HICH, blood-activating Chinese medicine should be the first choice.

The meta-analysis of this study still has some limitations. The meta-analysis of this study is a re-analysis based on all the initial studies, and the credibility depends on the data of the initial studies. The allocation concealment of 12 RCTs in the study is unknown, which may bias the results. There are differences in the number of trial samples in the 19 RCT studies included in this study, and the sample size of some trials is small, which will affect the accuracy of the results. The treatment cycle of the 19 RCT studies cannot be unified, which can cause errors in the adverse events of TCM treatment of HICH patients. The differences in the content and dosage of Chinese herbal medicines in the 19 RCT studies will also increase the heterogeneity of the results. All participants in this study are from China, and there is a lack of data from other countries and nationalities to prove the treatment effect. Therefore, larger-scale and sample-based clinical trials are needed to prove the efficacy of combined Chinese and Western medicine in the treatment of HICH patients.

## 6. Conclusion

There is drug resistance in Western medicine during the treatment of HICH, and the therapeutic effect is often limited. The results of this study showed that the efficacy of combined Chinese and Western medicine in the treatment of HICH is better than that of Western medicine alone. Furthermore, TCM is used as an adjunctive drug in clinical research of HICH to enhance the therapeutic effect of Western medicine.

However, due to the limited number of samples included in the study and certain limitations in the quality of some studies, the above conclusions still need to be further verified by large-scale and high-quality randomized double-blind controlled trials. In addition, when promoting the combined Chinese and Western medicine treatment in clinical practice, close attention should be paid to possible adverse reactions, and potential risks should be evaluated and managed in a timely manner to ensure the safety and feasibility of combined treatment.

## Author contributions

**Conceptualization:** Lixiaotian Zong, Desheng Zhou, Huan Dong, Chun Guo.

**Data curation:** Lixiaotian Zong, Yan Zeng, Xuqing Zhou.

**Formal analysis:** Lixiaotian Zong, Desheng Zhou, Yan Zeng, Zhong Li.

**Funding acquisition:** Chun Guo.

**Investigation:** Lixiaotian Zong, Xuqing Zhou.

**Methodology:** Lixiaotian Zong, Desheng Zhou, Huan Dong.

**Project administration:** Lixiaotian Zong.

**Resources:** Yan Zeng, Chun Guo.

**Software:** Lixiaotian Zong, Xuqing Zhou.

**Supervision:** Desheng Zhou, Zhong Li, Chun Guo.

**Validation:** Zhong Li, Huan Dong.

**Visualization:** Xuqing Zhou.

**Writing – original draft:** Lixiaotian Zong, Desheng Zhou, Yan Zeng, Zhong Li, Xuqing Zhou.

**Writing – review & editing:** Huan Dong, Chun Guo.

## Supplementary Material




