# Supplementary material for: Efficacy and safety of combined Chinese and Western medicine therapy for hypertensive intracerebral hemorrhage: A systematic review and meta-analysis of randomized controlled trials
Source: Medicine (Baltimore). 2025 Oct 10;104(41):e44632. doi: 10.1097/MD.0000000000044632 (PMC12517904; doi:10.1097/MD.0000000000044632)
Supplement: Supplementary file 1 [file medi-104-e44632-s001.docx]

| Outcome | Number of Studies | Sample Size | GRADE |
| --- | --- | --- | --- |
| Clinical effective rate | 15 | 1309 | High |
| NIHSS score | 7 | 586 | Moderate |
| GCS score | 4 | 706 | Moderate |
| Hs-CRP | 4 | 342 | Low |
| Adverse events | 5 | 72 | Low |
| Mortality | 1 | 68 | Very Low |
