# Supplementary material for: Efficacy and safety of combined Chinese and Western medicine therapy for hypertensive intracerebral hemorrhage: A systematic review and meta-analysis of randomized controlled trials
Source: Medicine (Baltimore). 2025 Oct 10;104(41):e44632. doi: 10.1097/MD.0000000000044632 (PMC12517904; doi:10.1097/MD.0000000000044632)

Figure S1. The funnel plots for the clinical efficacy of combined Chinese and Western medicine therapy for HICH.

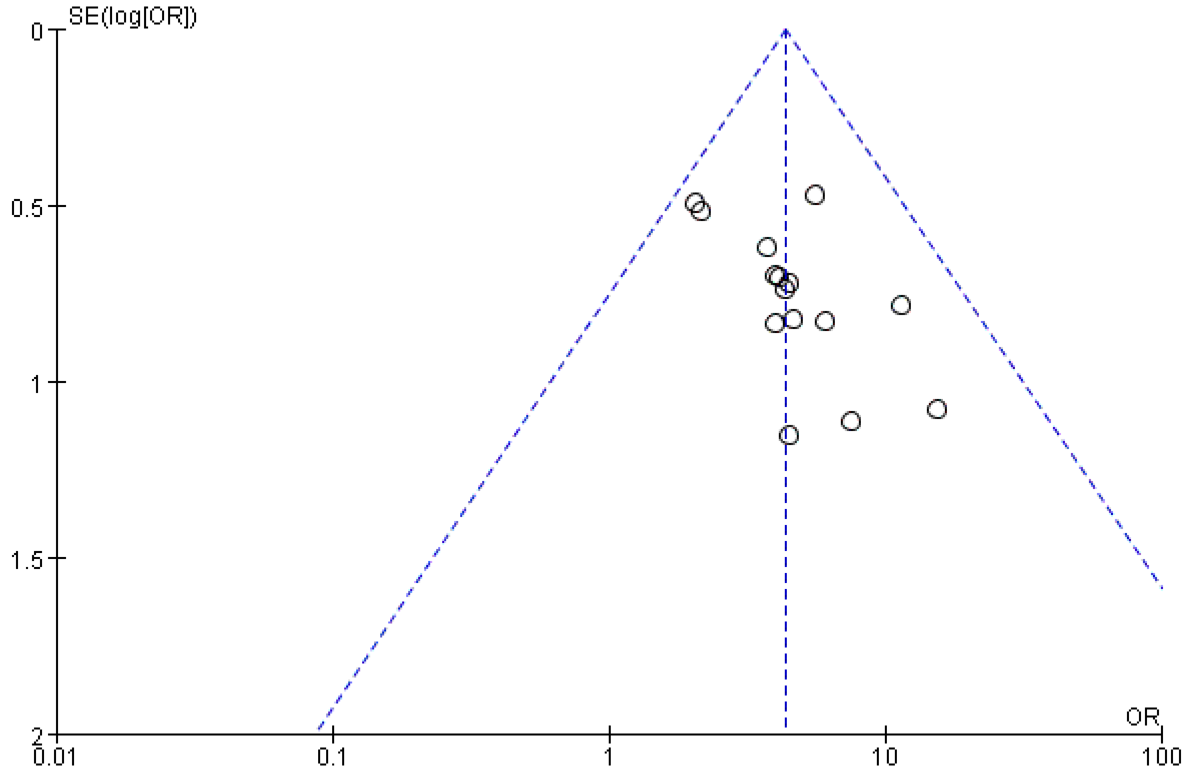

Supplement: Supplementary file 2 [file medi-104-e44632-s002.pdf]
